# Multivariate classification of livestock production systems in Mexico

**DOI:** 10.1007/s11250-025-04389-5

**Published:** 2025-03-22

**Authors:** Alejandro Zaldivar-Gomez, Beatriz Martínez-López, Gerardo Suzán, Oscar Rico-Chávez

**Affiliations:** 1 Departamento de Etología, Fauna Silvestre y Animales de Laboratorio, Facultad de Medicina Veterinaria y Zootecnia, Universidad Nacional Autónoma de México, Ciudad de Mexico, México; 2 Center for Animal Disease Modeling and Surveillance (CADMS), Department of Medicine and Epidemiology, School of Veterinary Medicine, University of California-Davis, Davis, CA USA

**Keywords:** Principal component analysis, Multivariate analysis, Cattle, Goat, Pig, Sheep, Production systems, Livestock production

## Abstract

The Mexican food production industry spans diverse agricultural and livestock products. Growing demand for animal-based products is driving significant changes in Livestock Production Systems (LPS), including shifts in location, herd sizes, and specializations. Global trade in livestock products has impacted Mexican producers’ competitiveness and natural resource demands, raising environmental concerns. Better understanding of production system variations can assist decision-makers in enhancing agricultural sustainability. The study aimed to characterize the different types and distribution of LPSs in Mexico and their key factors. A conceptual model was developed reflecting the elements and interactions within production systems for cattle, sheep, goats, and pigs. Input variables were defined using this model, and data were gathered from government and official sources. A Principal Component Analysis (PCA) and a Hierarchical Cluster on Principal Components (HCPC) were used to characterize LPSs and classify states based on this typology. The multivariate analysis identified four production profiles, and the country’s 32 states were classified into four distinct LPSs. The typology revealed by these production systems was consistent with traditional definitions previously established in Mexico. We observe changes within cattle and pig production systems, most notably in the adoption of new technology and integration of crop and livestock enterprises. Systemic heterogeneity was evident, with less economically developed states the most likely to display differences in the competitiveness of their production. This analysis represents the first quantitative synthesis of LPSs in Mexico and demonstrates the need for further investigation into their conditions and the factors that influence its diversification.

**Supplementary Information:**

The online version contains supplementary material available at 10.1007/s11250-025-04389-5.

## Introduction

The relationship between animal husbandry and agriculture is a fundamental aspect of global agriculture. Given the interrelated factors of livestock density, agricultural productivity, economic development, and growing environmental impact, it is crucial to quantify and characterize the interactions between agriculture and livestock (Thornton 2010; Robinson et al. 2011). A focus based on Livestock Production Systems (LPS) has been used to analyze these interactions and understand how agriculture and livestock production influence each other (Darnhofer et al. 2012). This approach is essential for the livestock sector because it can be used to analyze how LPS elements relate to poverty determinants (Dixon et al. 2001), strengthening production processes on farms (Darnhofer et al. 2010), measure livestock environmental risks, and estimate animal disease transmission risk (Grace et al. 2015). The latest LPS classifications are based on many characteristics and vary in usefulness, completeness, and ease of information acquisition (Robinson et al. 2011).

Mexico has a strong agricultural and livestock tradition; approximately 68.4% of its area (134.4 million hectares) is dedicated to livestock and agricultural production (SIAP 2023b). Mexico has a diverse climate, vegetation, culture, and social development, making LPS classification difficult. In the 1980s, the technology and production methods generated by research were not being adopted by producers due to their disconnection from the reality in the field (Pomareda and Vargas 1997). In colaboration with international partners, four regions of the country were delineated based on livestock practices and environmental factors: humid tropical, dry tropical, temperate, and arid-semiarid (Villegas-Duran et al. 2001; Amendola-Massiotti et al. 2005).

The classification of LPS in Mexico can be categorized according to the technology employed, market integration, production capacity, and geographic distribution of production practices (Espinosa-García, 2012; Salas-González et al. 2013; Palma-García, 2014). Over the past three decades, the competitiveness of Mexico’s livestock sector has declined due to overexploitation of the land, lack of financing and training for producers, the climate change, and the openness to international trade (Enríquez Quiroz et al. 2021; Montaño Méndez et al. 2021). Policymakers in Mexico should use LPS to develop adaptive public policies addressing challenges within the livestock sector.

Multivariate data analysis is essential for characterizing animal husbandry practices by revealing key patterns and relationships that shape production systems (Musafiri et al. 2020; Freitas Silveira et al. 2021). Dimension reduction techniques, such as Principal Components Analysis (PCA), Hierarchical Cluster Analysis (HCA), factorial analysis (FA), and Discriminant Analysis (DA), are widely used to investigate LPS (Vertel-Morinson et al. 2016; Makate et al. 2018; Shaner 2019). In Mexico, several research studies have employed multivariate analysis techniques in their investigation of LPS. (Figueroa-Sandoval et al. 2019; García-Villegas et al. 2020; Vélez Izquierdo et al. 2021). These studies utilize farm-level data, which requires significant effort to collect. Furthermore, studies that use farm-level data often focus on small regions and short time cycles, complicating the comparison of identified LPS (Fresco and Westphal 1988; Meuwissen et al. 2019; Verdouw et al. 2019). It’s plausible that livestock research programs haven’t started developing technological innovations for each system. Therefore, expanding the spatio-temporal scales may improve the results in defining a classification for LPS and provide a stronger baseline for policymaking and scientific research.

In this study, our aim was to generate a consistent classification of the LPS in Mexico using an exhaustive list of indicators within a multivariate analysis framework. We expanded the spatio-temporal scales to define a more consistent classification structure for LPS in Mexico, aiming to provide a stronger baseline for policymaking and scientific research.

## Materials and methods

### Model of the livestock production system

A representative model of the bovine, ovine, caprine, and porcine supply chains was employed to define LPS in Mexico and to guide the subsequent statistical analysis (Wadsworth 1997; ten Napel et al. 2011; Madry et al. 2013; Gaitán-Cremaschi et al. 2019; Shaner 2019). These livestock species were selected based on data availability and their importance in reflecting livestock production in Mexico.

**Fig. 1:**
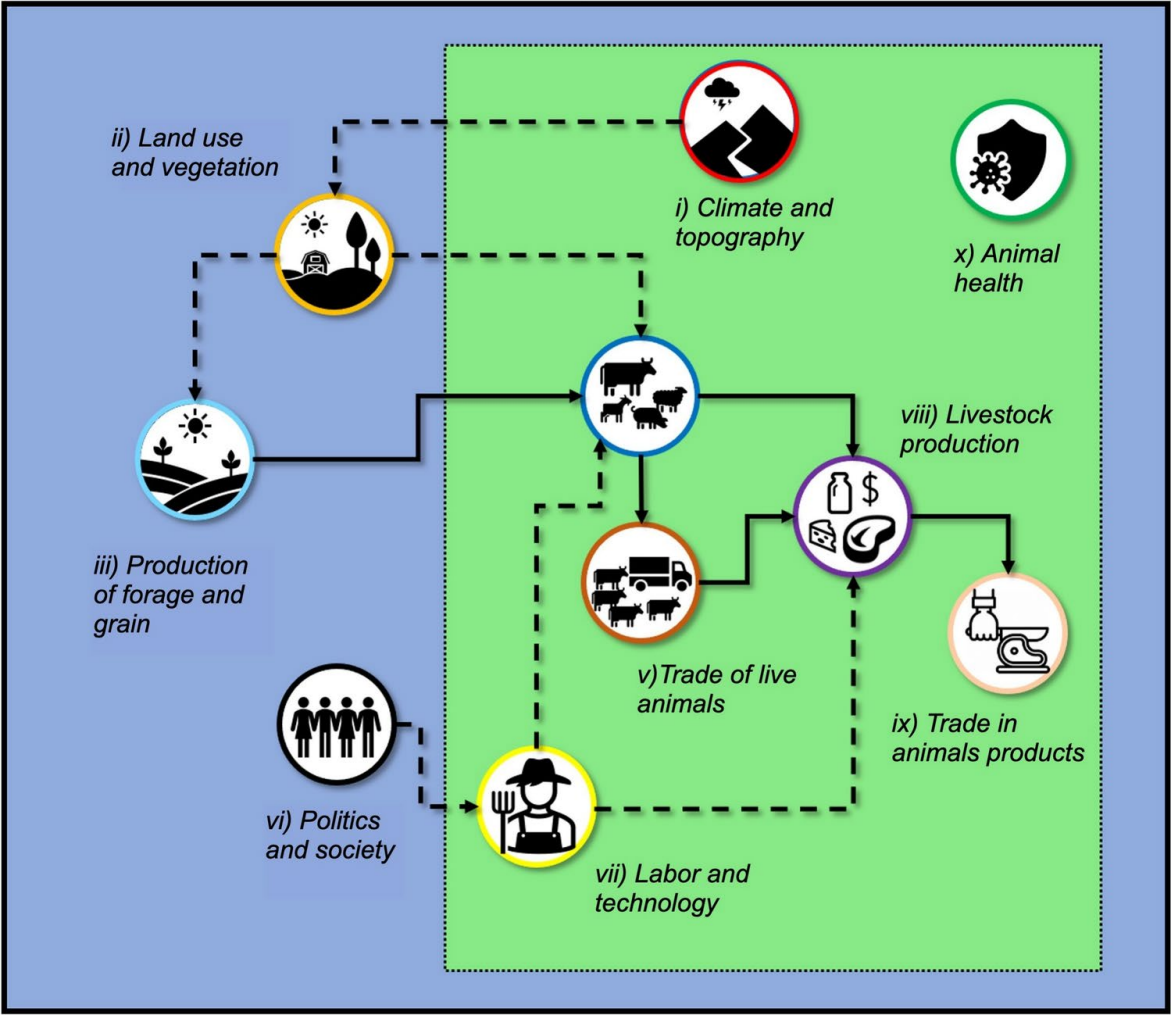
Model of the livestock production system (LPS) in Mexico. Solid arrows represent the flow of physical factors and broken arrows show the flow of information or interactions. Animal health actively regulates and interacts with all the elements enclosed in the green box. The blue box represents the entire LPS

The key elements for the model were selected considering the livestock management practices and environmental, economic, social and marketing factors associated with each supply chain. To identify these elements and their interrelationships, existing literature and reviews of the livestock sector in Mexico were consulted (Pomareda and Vargas 1997; Villegas-Duran et al. 2001; Amendola-Massiotti et al. 2005; Echavarría-Cháirez and Gómez-Ruiz 2013; Martínez-González et al. 2017; FAO 2019). As shown in Fig. 1, a framework was constructed to represent the key elements and their interrelationships, with emphasis placed on the hierarchies and dependencies that exist between them. Table 1 provides an overview of the key elements, their descriptions, and the corresponding sources of information.

**Table 1:** Key elements of the livestock production system model in Mexico, including their descriptions and sources of information

Variable	Description	Data source
i) Climate and topography	Environmental factors, such as temperature and rainfall, along with the physical landscape influencing livestock production	(INEGI 2013; SIAP 2023a)
ii) Land use and vegetation	Types of land allocated for agriculture and livestock, and the natural vegetation supporting grazing and feed production	(COTECOCA 2014; INEGI 2016, 2017)
iii) Production of forage and grain	Cultivation and availability of crops used as animal feed, essential for supporting livestock growth and health	(SIAP 2019b)
iv) Livestock density	The concentration of animals per unit area, which impacts resource use, management practices, and environmental sustainability	(PGN 2019)
v) Trade of live animals	The places where livestock are exchanged for breeding, sale, or export, influencing supply chains and market dynamics	(PGN 2020)
vi) Politics and society	Government policies, regulations, and social factors that shape livestock production systems and market operations	(FUNDAR 2019; INEGI 2019a, 2020; CONAPO 2020; SIAP 2023a)
vii) Labor and technology	The availability of skilled labor and the adoption of technological innovations in livestock production, affecting efficiency and productivity	(INEGI 2019a; SIAP 2023a)
viii) Livestock production	The output of animal-based products, such as meat, milk, and wool, reflecting the productivity and yield of livestock systems	(INEGI 2019b; SIAP 2020, 2021, 2023a)
ix) Trade in animal products	Data on livestock slaughter and production, serving as indicators of market demand for meat and other animal-based products	(SIAP 2019a)
x) Animal health	The management of livestock health through disease prevention, control measures, and veterinary care to ensure productivity and food safety	(INEGI 2019a; SENASICA 2019, 2020, 2021)

### Data collection

We collected official statistical indicators and government data from Mexico at the state level between 2015 and 2019 to quantify the elements. For a detailed list of variables, please refer to the Online Resource 1. This timeframe was selected to account for the delay in consolidation of data repositories and to mitigate potential uncertainties introduced by the impact of the COVID-19 pandemic on the Mexican livestock sector. The initial dataset had 105 variables. Following a correlation matrix analysis, we excluded 37 variables with less than 10 moderate correlations within the dataset (*Pearson’s r* < 0.5). The retrospective analysis of data related to productive activities in Mexico was conducted without the direct participation of humans or animals. Consequently, the involvement of an ethics committee was not required.

### Data analysis

We used descriptive statistics such as the mean, minimum, and maximum to explore the data. To set the production profiles, we applied a PCA. This method reveals the structure of a dataset and reduces its dimensions into new uncorrelated variables that explain as much variance as possible (De la Garza-Garcia et al. 2013). We included the 68 variables in the PCA and retained four principal components based on the scree plot, which explained 57.1% of the cumulative variance (D’agostino and Russell 2005). We applied an orthogonal rotation to minimize the number of variables that had high loadings on each factor. Finally, we interpreted the most characteristic traits of each component by examining the contribution levels of the variables along with the eigenvectors greater than 0.5 and less than −0.5. We assigned a descriptive name that reflects each production profile.

We applied a Hierarchical Cluster on Principal Components (HCPC) to develop a typology of LPS in the Mexican states. This technique combines PCA with HCA and has been previously used in research related to identifying typologies in LPS (Kassambara 2017; Zoma-Traoré et al. 2020; Mugumaarhahama et al. 2021). We constructed a dendrogram using Ward’s method with the HCPC. The optimal number of clusters is chosen based on a criterion that minimizes the increase in inertia between consecutive clusters. This criterion is determined between three and ten possible clusters (Husson et al. 2010; Kassambara 2017). The initial division was consolidated by the *k-means* algorithm using the number of clusters defined by the HCA (Ahmed et al. 2020). We evaluated clustering quality by examining the concordance between the clusters and the production profiles obtained from the PCA.

The mean of each dataset variable was calculated for each cluster and overall. A comparative analysis of the variables across clusters was then conducted to determine the degree of their differentiation. A hypothesis test (*v.test*) was used to assess whether the mean within a category was equal to the overall mean (Husson et al. 2017). A *v-test* value over 1.96 implies a statistically significant p-value below 0.05. The direction of the v-test sign shows whether the cluster mean is lower or greater than the overall mean, helping explain inter-cluster variances. To show what production profiles are the most associated with clusters, we identified the coordinates on the axes of each profile in clusters. All analyses and visualizations were done using FactoMineR (Lê et al. 2008) and Factoextra (Kassambara and Mundt 2020) packages implemented in the free software R (*v 4.0.3*) (R Core Team 2021).

## Results

### Principal component analysis

**Fig. 2:**
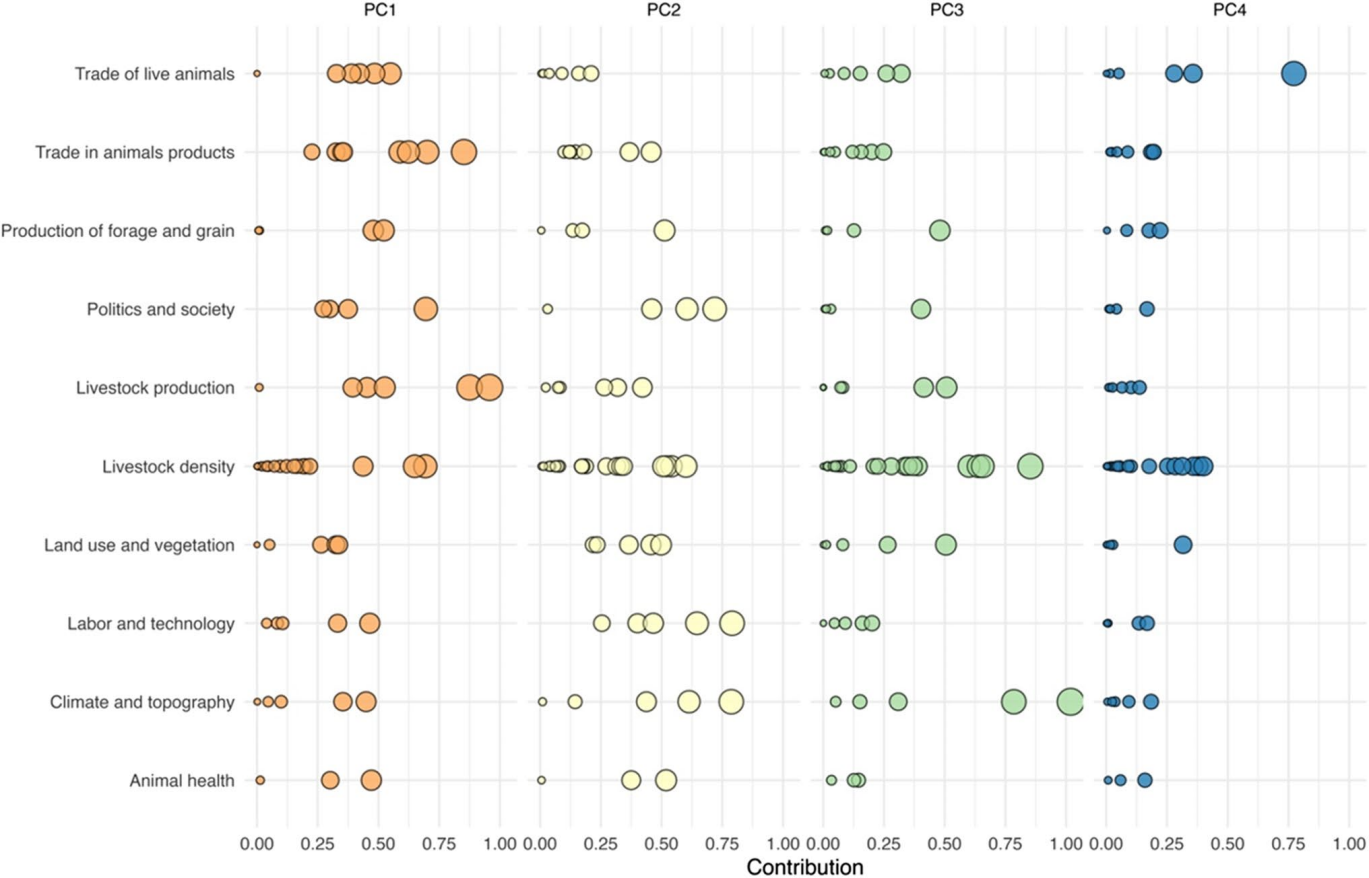
Contribution levels of variables to each principal component identified in the PCA. Each circle represents a variable, with its size and position reflecting the level of contribution to the respective component, grouped by the key elements of the proposed livestock production systems model

We identified four components based on the contribution of the variables to their formation and factor loadings. Descriptive names were assigned to each component, capturing their respective production profiles. The contribution levels of the variables are illustrated in Fig. 2, while the detailed dimensions and factor loadings from the PCA can be found in Online Resource 3.

The first livestock production profile (PC1) was named *“swine and bovine agroindustry”* and explained 19.02% of the total variance. This profile includes cattle, pigs, cereal, and forage crops and contributes significantly to the state’s GDP. Cattle production is characterized by dual-purpose and breeding cattle. The integration of production and commercialization is evident in the operations of slaughterhouses, feedlots, stockyards, and livestock fairs. Finally, the rural population is marked by poverty, with livestock husbandry representing their primary economic activity.

The *“intensive livestock” production profile (PC2)* explained 17.78% of the variance. This profile is characterized by a high density of dairy and beef cattle, achieved through the adoption of practices that enhance livestock production. Lower poverty rates indicate a better socioeconomic environment for stockbreeders. Additionally, farms receive technical assistance and use technology for livestock production. This profile has the capacity to facilitate significant advancements in epidemiological surveillance and animal health programs.

Profile three (PC3) represents *“small ruminants”* and accounts for 13.19% of the total variance. This profile is characterized by highland and temperate zones, which contribute to a high forage production yield. It includes ovine and caprine species raised for meat, milk, wool, and breeding, as well as pig fattening.

The fourth profile (PC4), "export of live cattle", accounted for 7.13% of the variance. This profile includes dairy and breeding cattle, especially those raised for reproduction. It also includes the trade of live animals, which encompasses stockyards and export pens for cattle.

### Clustering – characteristics of clusters

**Fig. 3:**
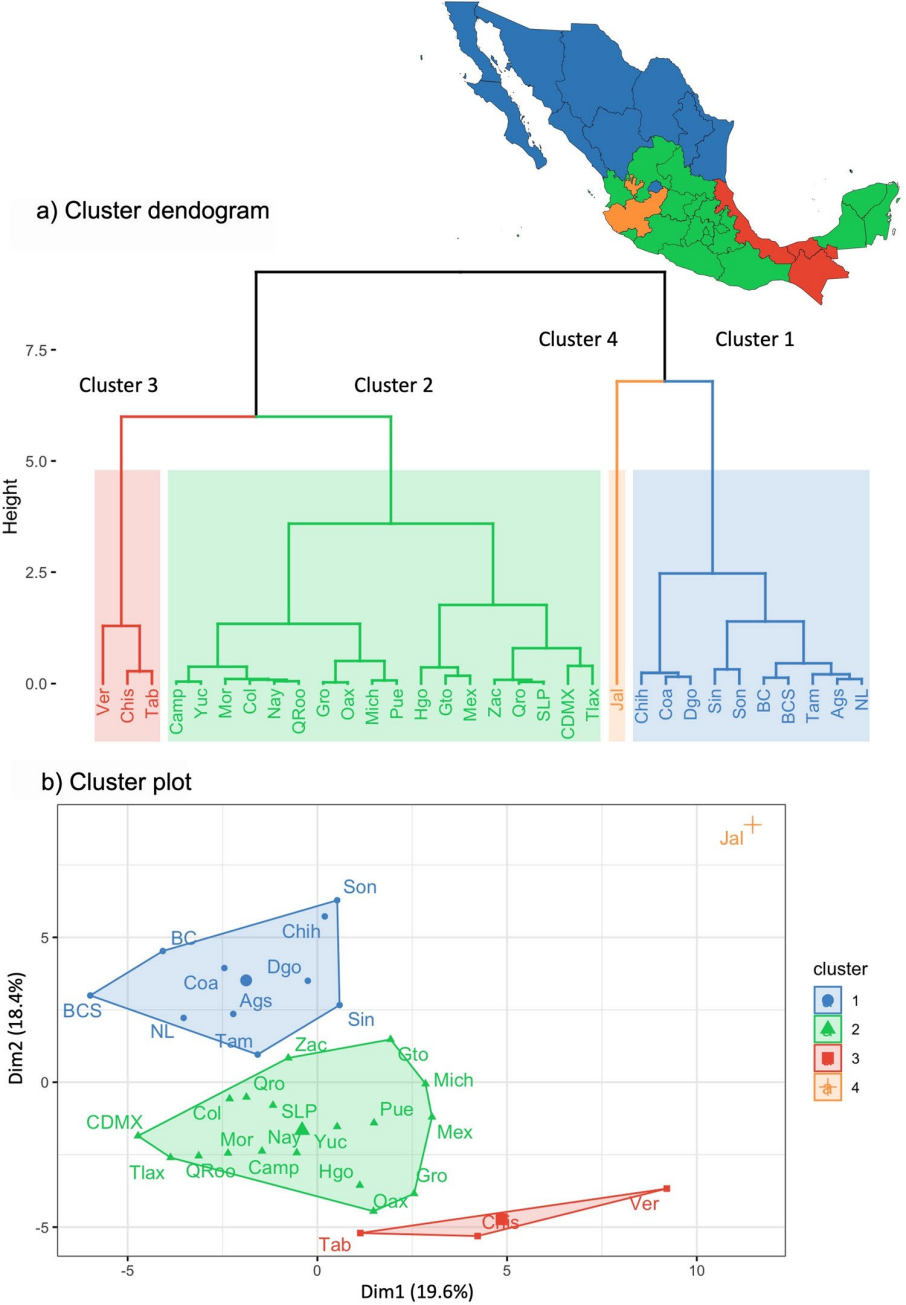
Principal components clustering of livestock production systems in Mexico. **a** Hierarchical clustering dendrogram based on the Ward criterion. Branch lengths are proportional to the degree of dissimilarity between groups. The solution of the groups is indicated in four colors. **b** Cluster plot showing the distribution of groups in the first and second principal components. The number of groups retained was established based on the dendrogram and the groups were consolidated using the *K-means* algorithm. Abbreviations: Chih: Chihuahua, Coa: Coahuila, Dgo: Durango, Sin: Sinaloa, Son: Sonora, BC: Baja California, BCS: Baja California Sur, Ags: Aguascalientes, NL: Nuevo León, Tam: Tamaulipas, Jal: Jalisco, Gro: Guerrero, Oax: Oaxaca, Col: Colima, Mor: Morelos, Camp: Campeche, Yuc: Yucatán, Nay: Nayarit, QRoo: Quintana Roo, Zac: Zacatecas, Qro: Querétaro, SLP: San Luis Potosí, CDMX: Ciudad de México, Tlax: Tlaxcala, Hgo: Hidalgo, Mich: Michoacán, Pue: Puebla, Gto: Guanajuato, Mex: Estado de México, Ver: Veracruz, Chis: Chiapas, Tab: Tabasco

The dendrogram in Fig. 3a suggests a solution of four clusters (C1, C2, C3, and C4) of states with a typology well-differentiated from LPS. Cluster 1 (C1) consists of 10 states (31.3% of the total), Cluster 2 (C2) includes 18 states (56.3%), Cluster 3 (C3) comprises 3 states (9.4%), and Cluster 4 (C4) is represented by 1 state (3.0%). This distribution highlights the inherent heterogeneity of LPS across the country, with smaller clusters representing states with unique or distinct production characteristics.

The separation was confirmed using *K-means* algorithm with a predefined group number of four. The loadings of the states grouped by *K-means* are shown in Fig. 3b, confirming that all states retained the same assignments as in the hierarchical clustering, indicating consistency and robustness in the cluster definition.

The comparative analysis of the variables across clusters showed significant differences between the cluster-specific means and the overall mean (*P* < 0.05). The number of variables with statistical significance was variable among the clusters. C4 had the highest number of variables with 21, followed by C3 with 16, C1 with 15, and C2 with 10 variables. Online Resource 3 presents the variables for each cluster, along with their corresponding descriptive statistics.

### Cluster 1 (C1) Intensive and high investment production system

The Cluster 1 is composed of 10 northern states of Mexico, showed high coordinate on axis PC2 (*v.test* = 3.79, *P* < 0.001) and low coordinates with axe PC1 (*v.test* = −1.96, *P* < 0.05).

### Cluster 2 (C2) Semi-extensive, traditional, and low-input production system

The Cluster 2 is composed of 18 states that scored high on axis PC3 (v.test = 2.80, *P* < 0.01) and low on axis PC2 (v.test = −3.01, *P* < 0.01).

### Cluster 3 (C3) States with extensive and low infrastructure production systems with vulnerability to poverty

Cluster 3 is composed of three states with high coordinates on axis PC1 (v.test = 2.41, *P* < 0.05) and low coordinates on axes PC2 (v.test = −2.43, *p* < 0.05) and PC3 (v.test = −2.51, *P* < 0.05).

### Cluster 4 (C4) State with traditional and agroindustrial production systems

Cluster 4 is comprised solely of Jalisco, which exhibits high coordinates on axes PC1 (v.test = 3.19, *P* < 0.01) and PC2 (v.test = 2.55, *P* < 0.05).

## Discussion

This study represents the first multivariate analysis that proposes an integrated approach to classify LPS in Mexico. It emphasizes the intrinsic interconnectivity and dynamism of production practices (Cadillo-Benalcazar et al. 2020; Dutilly et al. 2020). This approach incorporates official quantitative variables that represent the elements of productive activity across different livestock species, based on a five-year longitudinal study, and provides a more comprehensive understanding of the dynamics within the livestock sector (ten Napel et al. 2011; Ghimire et al. 2021; Sekaran et al. 2021). Our study identified four production profiles that define the typology of LPS in Mexico, categorizing the country’s states into four distinct regions.

By employing PCA, HCPC, and *K-means* algorithm within a multivariate analysis, we successfully defined the typology of LPS and effectively grouped states accordingly. A significant challenge was the analysis of a dataset with high dimensionality, which carries the risk of overfitting and complicates the relationships between variables (Pires and Branco 2019). Due to the limitations of the sample size and data availability, we employed a state-level aggregation approach. We applied PCA to reduce the dimensionality of the data, enabling us to prioritize the most pertinent variables and identify significant patterns (Johnstone and Paul 2018). This approach provided the basis for subsequent analyses employing advanced techniques (Feng et al. 2012; Ayesha et al. 2020).

Our typology of LPS aligns with previous research conducted in Mexico, including studies on geographic distribution, market integration, technology adoption, and socioeconomic outcomes (Escareño-Sánchez et al. 2011; Vázquez-Martínez et al. 2018; González-Padilla et al. 2019; Rangel et al. 2020; Angel-Hernández et al. 2021; Chávez-Espinoza et al. 2022). This alignment validates our findings and elucidates the integration of production profiles within the suggested livestock production framework in the country.

Our analysis revealed two main production profiles in Mexico: the *“swine and bovine agroindustry”* profile (PC1) and the *“intensive livestock”* profile (PC2). The PC1 profile reflects an evolution of the traditional LPS into more regionally integrated agro-industrial models, while PC2 represents a more distinct shift away from traditional practices, driven by technological advancement, globalization, private investment, and production optimization (Kay 2009; Ayala-Garay et al. 2011; Barros and Martínez-Zarzoso 2022). Despite these inherent differences, our results demonstrate that both profiles fluctuate within the identified LPS typologies (C1, C3, and C4), sometimes being dominated by one profile while at other times displaying a significant combination of both.

The technological disparity between northern (C1) and southern (C3) Mexico is well-documented. However, our analysis provides a detailed examination of the driving factors that contribute to these disparities, highlighting infrastructure as a key driver for the adoption of advanced technologies (Ruiz-Torres et al. 2022).

This is particularly evident in the *intensive and high investment production system* (C1) of the northern states, which employs infrastructure to facilitate large-scale production and improve access to global markets. Despite the economic efficiency of these technologically advanced systems, they face significant challenges in environmental sustainability due to their dependence on intensive inputs (Murray-Tortarolo and Jaramillo 2019; Theusme et al. 2021).

On the other hand, the *extensive and low infrastructure production systems* (C3) of the southern states, including Veracruz, Chiapas, and Tabasco, have markedly low due to the presence of poor conditions and limited technology (Lassala et al. 2020; Villarroel-Molina et al. 2021). However, these systems demonstrate resilience to economic crises by serving as suppliers of calves for finishing (Skaggs et al. 2004; Rangel et al. 2020), while also engaging in diversification and traditional practices such as concentrate supplementation and crossbreeding (Lizbeth et al. 2021). Furthermore, our analysis corroborates the expansion of larger feedlots in this region, a salient trend that has emerged in recent years and indicates a substantial transformation in the region (Bautista-Martínez et al. 2020).

Jalisco (C4) is unique in that it shows a significant combination of both profiles where livestock industrialization, driven by investment in technology, coexists with the low socio-economic conditions faced by rural communities (Jiménez-Jiménez et al. 2019). This duality reflects the continuity between traditional practices and modern intensification, where the incorporation of technology has improved productivity in cattle and swine, supported by evidence from various studies in Mexico (Castillo Girón and Ayala Ramírez 2022; Gamboa-Chavarría et al. 2023).

Our findings indicate that this trend in livestock production is likely to continue, resulting in increased competitiveness for some producers while others may become more vulnerable (Salinas-Martínez et al. 2020; Faisal et al. 2021). It is evident that there is a necessity for the implementation of targeted public policies that invest in infrastructure and promote technology adoption in accordance with the specific needs of each region.

Cluster C2 is divided into two distinct regions with differing livestock production characteristics, both heavily influenced by climatic conditions. The first region, located in the central plateau, is predominantly focused on sheep farming, but faces significant challenges due to limited infrastructure and poverty, leading to subsistence-level operations. The semi-arid climate with seasonal rainfall in this region strongly influences production. Sheep, resilient to these conditions, thrive on extensive grazing and low-quality forage.

The second region, covering the Pacific Basin and Yucatán Peninsula, supports both sheep and goats, as well as cattle and pigs. Goats can thrive in arid and tropical climates under traditional grazing systems that utilize diverse and often degraded vegetation. However, prolonged dry periods and climatic seasonality significantly limit productivity (Abizaid and Coomes 2004; Trilleras et al. 2015). These differences highlight the diverse production practices and challenges within C2, shaped by both socioeconomic factors and the reliance of sheep and goat systems on natural resources. This results in variable productivity and susceptibility to environmental variability.

The non-significant concordance of the PC4 (‘live cattle export’) production profile with any cluster is possibly due to its low eigenvalue, as there are not enough variables contributing to system differentiation at the state level. In fact, the northern region of Mexico, including states from C1, is the largest exporter of live cattle to the United States (SIAP 2021).

When interpreting the findings, it is important to consider the following constraints. The first constraint relates to the limitations of collecting information at the state level and should therefore be used with caution when making decisions about individual farms (Jones et al. 2017; Kaur et al. 2021). This analysis offers cross-sectional information and is not intended to evaluate changes in long-term production characteristics. The second limitation is the potential exclusion of data from backyard farms, which are not adequately represented in our analysis, despite contributing between 20 and 30% of national livestock production. It is important to note that Mexico does not have enough official sources of information dedicated to collecting data on these backyard farms (Cavallotti-Vázquez et al. 2013; Álvarez-Macías and Santos-Chávez, 2019; Guevara-Hernández et al. 2023).

Finally, the clustering analysis revealed that Cluster 3 (C3) and Cluster 4 (C4) comprise a small number of states, reflecting the inherent heterogeneity of the data rather than methodological limitations. These clusters represent states with unique characteristics that distinguish them significantly from the rest, justifying their classification into smaller groups. While this may raise concerns about sensitivity, it is common in clustering analyses to capture natural patterns in the data rather than enforce uniform distributions. Despite their small size, these clusters provide valuable insights into distinctive regional dynamics, which are critical for targeted interventions or policy development.

Our study updated the national Mexican LPS typology, emphasizing their characteristics. The objective was to provide decision-makers with reliable evidence and develop public policies that align with the national agricultural and livestock development agenda. This research aids future assessments of LPS trends and changes.

## Supplementary Information

Below is the link to the electronic supplementary material.

Supplementary file1 (XLSX 16.1 KB)

Supplementary file2 (XLSX 12.6 KB)

Supplementary file3 (XLSX 17.7 KB)

## Data Availability

The datasets analyzed during the current study are available in the LivestockProductionMexico repository, located at https://github.com/alexzaldivar/LivestockProductionMexico.git.
